# Associations of serum cystatin C concentrations with total mortality and mortality of 12 site-specific cancers

**DOI:** 10.3389/fmolb.2024.1209349

**Published:** 2024-04-25

**Authors:** Changzhi Huang, Jiayi Lu, Jing Yang, Zhenling Wang, Dong Hang, Zan Fu

**Affiliations:** ^1^ Department of General Surgery, The First Affiliated Hospital of Nanjing Medical University, Nanjing, China; ^2^ Department of Epidemiology, School of Public Health, Nanjing Medical University, Nanjing, China; ^3^ Jiangsu Key Lab of Cancer Biomarkers, Prevention and Treatment, Collaborative Innovation Center for Cancer Medicine, Nanjing Medical University, Nanjing, China

**Keywords:** cancer, cystatin C, mortality, prospective cohort study, UK Biobank

## Abstract

**Purpose:**

Cystatin C (CysC), beyond its biomarker role of renal function, has been implicated in various physical and pathological activities. However, the impact of serum CysC on cancer mortality in a general population remains unknown. We aimed to examine the associations of serum CysC concentrations with total mortality and mortality of 12 site-specific cancers.

**Methods:**

We included 241,008 participants of the UK Biobank cohort with CysC measurements who had normal creatinine-based estimated glomerular filtration rates and were free of cancer and renal diseases at baseline (2006–2010). Death information was obtained from the National Health Service death records through 28 February 2021. Multivariable Cox proportional hazards models were used to compute hazard ratios (HR) per one standard deviation increase in log-transformed CysC concentrations and 95% confidence intervals (95% CI) for mortality.

**Results:**

Over a median follow-up of 12.1 (interquartile range, 11.3–12.8) years, 5,744 cancer deaths occurred. We observed a positive association between serum CysC concentrations and total cancer mortality (HR = 1.16, 95% CI: 1.12–1.20). Specifically, participants with higher serum CysC concentrations had increased mortality due to lung cancer (HR = 1.12, 95% CI: 1.05–1.20), blood cancer (HR = 1.29, 95% CI: 1.16–1.44), brain cancer (HR = 1.19, 95% CI: 1.04–1.36), esophageal cancer (HR = 1.20, 95% CI: 1.05–1.37), breast cancer (HR = 1.18, 95% CI: 1.03–1.36), and liver cancer (HR = 1.49, 95% CI: 1.31–1.69).

**Conclusion:**

Our findings indicate that higher CysC concentrations are associated with increased mortality due to lung, blood, brain, esophageal, breast, and liver cancers. Future studies are necessary to clarify underlying mechanisms.

## 1 Introduction

Cystatin C (CysC) is a secreted cysteine protease inhibitor abundantly expressed in body fluids (Xu et al., 2015). Due to its relatively small molecular weight (∼13.3 kDa) and easy detection, CysC is commonly used in hospitals to measure the glomerular filtration rate (GFR) as an index of kidney function (Inker et al., 2012; Shlipak et al., 2013). However, emerging functional evidence suggests that CysC is directly involved in various physical and pathological activities beyond its renal function biomarker role. For example, CysC has shown the potential to regulate immune response (Pierre and Mellman, 1998), apoptosis (Mori et al., 2016), autophagy (Wang M. et al., 2021), and tumor metastasis (Kopitz et al., 2005) independently or through a potent inhibition of cysteine cathepsins. Therefore, variation in CysC levels may have additional clinical implications that warrant further investigation (Sarnak et al., 2005).

Several studies have investigated the association between circulating CysC concentrations and cancer prognosis, primarily among patients already diagnosed with malignancies, such as lung cancer (Chen et al., 2011), colorectal cancer (Kos et al., 2000), breast cancer (Decock et al., 2008), and prostate cancer (Perez-Cornago et al., 2020). Much less is known about the association in the general population, particularly those with normal renal function. Although there is evidence linking higher CysC levels to increased total cancer mortality according to the Cardiovascular Health Study (Fried et al., 2005) and the Whitehall Study (Emberson et al., 2010), the association was not replicated in two other cohort studies (Shlipak et al., 2006; Wu et al., 2010). Moreover, few studies have performed dose–response analysis or evaluated the association between CysC concentrations and site-specific cancer mortality.

In this context, leveraging data from the UK Biobank, a large prospective cohort study, we aimed to determine the association between serum CysC concentrations and mortality from common cancers among the general population. This study would improve our knowledge about the impact of circulating CysC on cancer mortality and provide novel biochemical support for the prognostic assessment of specific cancers. Such insights are crucial for developing effective strategies to reduce the risk of cancer-related deaths.

## 2 Materials and methods

### 2.1 Study population

The UK Biobank is a large prospective cohort study consisting of about half a million participants (aged 37–73 years) recruited between 2006 and 2010 across the United Kingdom (Collins, 2012). Sociodemographic, lifestyle, and health-related information was collected through self-reported questionnaires at the baseline assessment. A series of biological samples, including blood, were collected from participants to study biochemical and cellular markers (Elliott and Peakman). The ethical approval was obtained from the North West Multi-center Research Ethics Committee (11/NW/0382; 16/NW/0274), and all participants provided informed consent.

**FIGURE 1:**
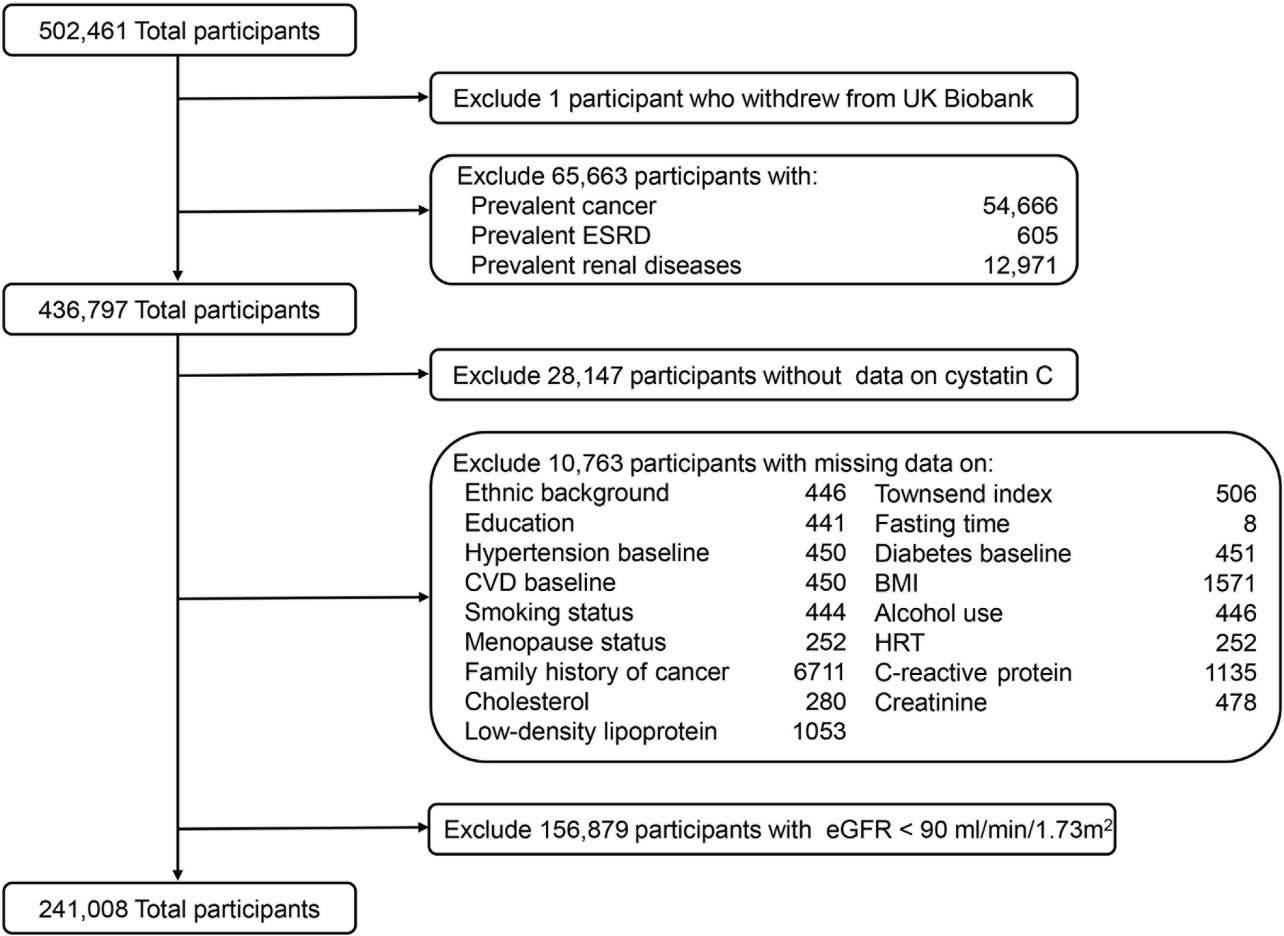
Flowchart of study participants from UK Biobank. ESRD, end-stage renal disease; CVD, cardiovascular diseases; BMI, body mass index; HRT, hormone replacement therapy; eGFR, estimated glomerular filtration rate.

In the current study, we excluded participants who had a history of cancer or renal disease before baseline according to electronic health records and self-reported answers at baseline (n = 65,663). Furthermore, participants who had missing data on serum CysC (n = 28,147) or main covariates were further removed (n = 10,763). To minimize reverse causality, we also excluded those with creatinine-based estimated glomerular filtration rates (eGFR) < 90 mL/min/1.73 m^2^, which is considered abnormal renal function (n = 156,879) (Stevens et al., 2013). Finally, 241,008 participants were included in the analysis (Figure 1).

### 2.2 Biomarker measurements

An immuno-turbidimetric assay based on the Siemens Advia 1800 platform (Siemens plc) was used to measure serum CysC concentrations. The average coefficients of variation (CV) in the low- and high-level internal quality control samples of CysC were 1.36% and 0.75%, relatively. Moreover, an external quality assurance scheme was conducted to verify the assay performance, showing that 100% of participated distributions (n = 20) were good or acceptable. In addition, serum creatinine and cholesterol concentrations were measured by enzymatic methods. C-reactive protein (CRP) concentrations were measured by an immuno-turbidimetric assay. Low-density lipoprotein (LDL) concentrations were measured by an enzymatic selective protection assay. Details about serum biomarker measurements and assay performances have been described in the online UK Biobank Showcase (http://biobank.ndph.ox.ac.uk/showcase/showcase/docs/serum_biochemistry.pdf).

The eGFR was calculated based on creatinine using the Chronic Kidney Disease Epidemiology Collaboration (CKD-EPI) equation (20). CKD-EPl equation expressed as a single equation: GFR = 141 × min (Scr/κ, 1)^α^ × max (Scr/κ, 1)^−1.209^ × 0.993^Age^ × 1.018 (if female) × 1.159 (if black). Scr is standardized serum creatinine in mg/dL, κ is 0.7 for females and 0.9 for males, *α* is −0.329 for females and −0.411 for males, min indicates the minimum of Scr/κ or 1, and max indicates the maximum of Scr/κ or 1. Normal creatinine-based estimated glomerular filtration rates were defined as greater than or equal to 90 mL/min/1.73 m^2^ (CKD Work Group, 2024).

### 2.3 Covariate assessment

Information on age, sex, ethnicity, fasting status, education degree, lifestyle factors (smoking status and alcohol consumption), and medical history (medical conditions, family history of cancer, and for women, menopausal status and ever use of hormone replacement therapy) was extracted from a self-reported questionnaire at baseline. Height and body weight were measured by trained health workers, and the body mass index (BMI) was calculated as weight in kilograms divided by height in meters squared (kg/m^2^). The Townsend deprivation index, an indicator of socioeconomic status, was derived from data on unemployment, household overcrowding, non-home ownership, and non-car ownership (Jarman et al., 1991). Physical activity was measured as total metabolic equivalent task (MET)-hours per week for all activity, including walking, moderate, and vigorous activity (Ainsworth et al., 1993).

### 2.4 Ascertainment of cancer deaths

Death certificates were obtained from the National Health Service Information Centre (England and Wales) and the National Health Service Central Register Scotland (Scotland). The 10th revision of the World Health Organization’s International Statistical Classification of Diseases (ICD-10) diagnosis codes was used to ascertain the primary cause of death. Total cancer (C00-D48) and the 12 most common cancers in the UK Biobank were assessed, which included lung cancer (C34), colorectal cancer (C18-C20), pancreatic cancer (C25), blood cancer (C81-C96), brain cancer (C71), esophageal cancer (C15), breast cancer (C50), liver cancer (C22), prostate cancer (C61), ovarian cancer (C56), stomach cancer (C16), and kidney cancer (C64) (Supplementary Table S1).

### 2.5 Statistical analysis

The follow-up time was calculated from the date of recruitment to the date of death, loss to follow-up, or the last follow-up (28 February 2021). Cancer mortality rates pertained to the number of deaths from a specific site-related cancer per a specific number of person-years of follow-up. Multivariable-adjusted restricted cubic splines with five knots (the 5th, 27.5th, 50th, 72.5th, and 95th percentiles) were used to plot the dose–response relationship between serum CysC concentrations and cancer mortality. A likelihood ratio test was used to compare the model with both the linear and the cubic spline terms, with *P* for nonlinear <0.05 considered nonlinearity and *P* for nonlinear >0.05 & *P* for linear <0.05 denoting linearity. Cox proportional hazard models with age as the time scale were used to calculate hazard ratios (HR) and 95% confidence intervals (CI) for cancer mortality according to quintiles and per one standard deviation (SD) increment of the log-transformed CysC concentrations. The proportional hazard assumption was based on Schoenfeld residuals, and no violation was found in this study (Richmond et al., 2019). Model 1 was adjusted for age at baseline assessment (years), sex (female, male), ethnicity (White, not White), and fasting status (yes, no). Model 2 was additionally adjusted for the Townsend index (continuous), college or university degree (yes, no), BMI (kg/m^2^), smoking status (never, previous, current), pack-years of smoking (continuous), alcohol consumption (never, special occasions only, 1–3 times per month, 1–2 times per week, 3–4 times per week, daily/almost daily), physical activity (MET-hours/week), family history of cancer (yes, no), prevalent hypertension (yes, no), diabetes (yes, no), cardiovascular diseases (CVD) (yes, no), and for women, menopausal status (yes, no) and ever use of hormone replacement therapy (yes, no). Model 3 was further adjusted for serum cholesterol (mmol/L), LDL (mmol/L), CRP (mg/L), and creatinine-based eGFR (mL/min/1.73 m^2^).

Stratified analyses were conducted according to age at blood drawn (<55; ≥55 years), sex (male; female), BMI (<30; ≥30 kg/m^2^), and smoking status (non-smoker; smoker). Sensitivity analyses were performed by excluding people who died within 2 years or had unfavorable self-assessment of overall health at baseline. Two-sided *p-*values less than 0.05 were considered statistically significant. All the statistical analyses were performed using SAS version 9.4 (SAS Institute, Cary, NC).

## 3 Results

During a total of a total of 2,860,841 person-years of follow-up (median follow-up: 12.1 years; interquartile range: 11.3–12.8 years), 5,744 of 241,008 participants died from cancer. Table 1 describes the baseline characteristics of participants according to quintiles of serum CysC concentrations. Participants with higher CysC concentrations were more likely to be older, males, current smokers, and have a higher Townsend deprivation index and BMI. In addition, they tended to have prevalent hypertension, diabetes, and CVD; they also had higher levels of CRP and LDL. The baseline characteristics of the subjects, stratified based on whether cancer death occurred, are presented in Supplementary Table S2.

**TABLE 1:** Baseline characteristics of UK Biobank participants with normal creatinine-based eGFR by quintile of serum cystatin C concentration^a^.

Characteristics	Quintile of CysC concentration, mg/L				
	Q1 (0.36–0.76)	Q2 (0.76–0.82)	Q3 (0.82–0.87)	Q4 (0.87–0.94)	Q5 (0.94–4.19)
Participants, No.	48,123	48,491	47,674	48,378	48,342
Age at assessment, year	51.1 (7.5)	53.1 (7.8)	54.2 (7.8)	55.3 (7.8)	56.6 (7.6)
Female, %	77	61	51	44	37
White race, %	92	93	94	94	93
College or university degree, %	40	38	36	33	28
Fasting when blood drawn, %	4	4	4	5	6
Townsend deprivation index	−1.4 (3.0)	−1.4 (3.0)	−1.4 (3.1)	−1.2 (3.1)	−0.7 (3.3)
Body mass index, kg/m^2^	25.2 (3.9)	26.2 (4.2)	27.0 (4.4)	27.8 (4.7)	29.4 (5.7)
Physical activity, MET hour/week	39.9 (31.6)	39.9 (31.9)	39.4 (31.9)	39.2 (32.4)	37.7 (31.9)
Smoking status, %^b^					
Never	62	59	57	53	46
Previous	31	32	32	33	32
Current	7	8	10	14	22
Alcohol consumption, %^b^					
Daily or almost daily	21	21	21	21	18
Three or four times a week	26	26	24	23	19
Once or twice a week	27	26	26	26	25
One to three times a month	11	11	11	11	12
Special occasions only	10	10	10	11	14
Never	6	7	7	8	11
Prevalent hypertension, %	15	19	22	26	33
Prevalent diabetes, %	4	4	4	5	7
Prevalent CVD, %	2	3	3	4	7
Postmenopausal, %^c^	28	30	30	28	26
Ever HRT use, %^c^	18	18	18	17	16
Family history of cancer, %	32	33	34	35	35
eGFR, mL/min/1.73 m^2^	103.8 (7.3)	100.9 (6.6)	99.5 (6.2)	98.3 (5.9)	97.0 (5.5)
C-reactive protein, mg/L	1.73 (3.26)	1.98 (3.55)	2.25 (3.86)	2.61 (4.14)	3.68 (5.42)
Cholesterol, nmol/L	5.61 (1.06)	5.71 (1.09)	5.76 (1.11)	5.76 (1.13)	5.67 (1.18)
Low-density lipoprotein, nmol/L	3.42 (0.81)	3.54 (0.83)	3.61 (0.85)	3.64 (0.86)	3.61 (0.89)

^a^ Normal creatinine-based eGFR was defined by the CKD-EPI as ≥90 mL/min/1.73 m2. Values are expressed as means (SD) unless otherwise indicated.

^b^ The total did not sum to 100% because a small proportion of participants chose “prefer not to answer".

^c^ Among women only.

Abbreviations: eGFR, estimated glomerular filtration rate; CysC, cystatin C; MET, metabolic equivalent task; CVD, cardiovascular disease; HRT, hormone replacement therapy; CKD-EPI, Chronic Kidney Disease Epidemiology Collaboration; SD, standard deviation.

**FIGURE 2:**
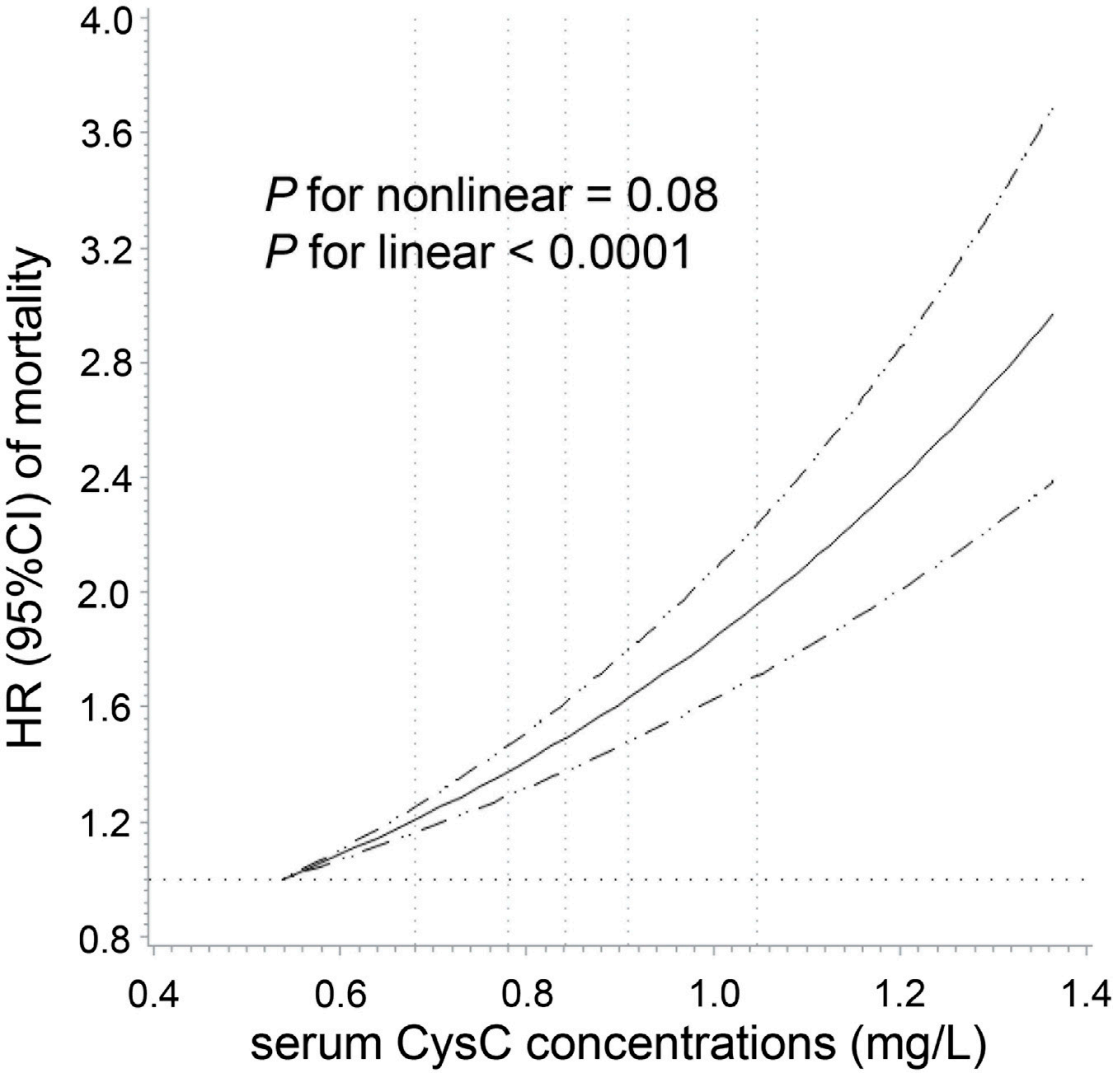
Dose–response association of serum cystatin C concentrations with total cancer mortality among participants with normal creatinine-based eGFR. Multivariable Cox regression models with restricted cubic spline analysis were performed, adjusting for the same set of covariates as in Model 3. Cystatin C concentrations above 99.9% and below 0.1% were not plotted due to wide confidence intervals at the extremes. The solid line represents estimates of hazard ratio (HR), and the dashed lines represent 95% confidence intervals (CI). The dashed lines perpendicular to the horizontal axis represent the 5th, 27.5th, 50th, 72.5th, and 95th percentiles of cystatin C, respectively. The dashed line perpendicular to the vertical axis represents the HR equal to 1. Normal creatinine-based eGFR was defined by the CKD-EPI as ≥90 mL/min/1.73 m^2^.

**FIGURE 3:**
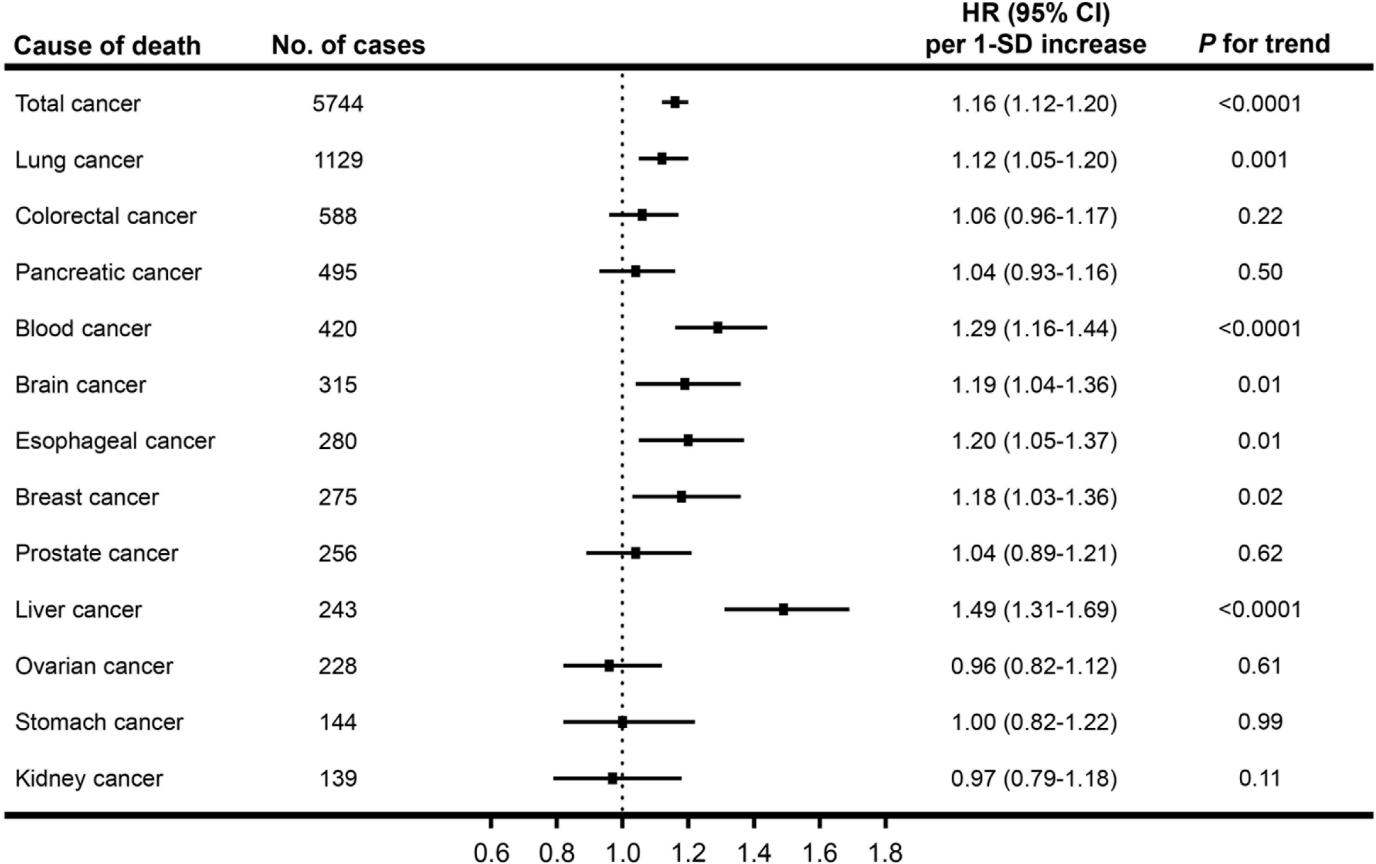
Associations of serum cystatin C concentrations and cancer mortality. Multivariable Cox proportional hazard models were used to calculate hazard ratios (HR) and 95% confidence intervals (CI) for cancer mortality per one standard deviation (SD) increment of the log-transformed cystatin C concentrations.

We observed a positive linear relationship between CysC concentrations and total cancer mortality among participants with normal kidney function (*P* for linear <0.0001) after adjustment for sociodemographic information, lifestyle factors, medical history, specific biomarkers, and renal function (Figure 2). As shown in Figure 3, a per 1-SD increment of the log-transformed CysC concentrations was associated with a 16% higher risk of total cancer mortality (HR = 1.16, 95% CI: 1.12–1.20) in Model 3. In the site-specific analysis (Figure 3), CysC was positively associated with mortality from lung cancer (HR = 1.12, 95% CI: 1.05–1.20), blood cancer (HR = 1.29, 95% CI: 1.16–1.44), brain cancer (HR = 1.19, 95% CI: 1.04–1.36), esophageal cancer (HR = 1.20, 95% CI: 1.05–1.37), breast cancer (HR = 1.18, 95% CI: 1.03–1.36), and liver cancer (HR = 1.49, 95% CI: 1.31–1.69). Multivariable restricted cubic spline analysis showed that CysC had positive linear associations with mortality from the above-mentioned cancer types (*P* for linear <0.05) (Supplementary Figure S2). However, the associations were non-statistically significant between CysC and mortality from the other types of cancer located at the colorectum, pancreas, prostate, ovarian, stomach, and kidney. The HRs and 95% CIs for mortality according to quintiles of CysC concentrations are presented in Supplementary Table S3. When compared to the lowest quintile, individuals in the highest quintile exhibit a heightened mortality risk for lung cancer (HR = 1.36, 95% CI: 1.23–1.51), blood cancer (HR = 1.54, 95% CI: 1.05–2.26), and liver cancer (HR = 2.46, 95% CI: 1.45–4.17) after adjusting for relevant confounding variables.

In the stratified analyses by age, gender, BMI, and smoking status, the associations of serum CysC concentrations with total and site-specific cancer mortality were generally similar across subgroups despite several exceptions (Supplementary Figures S2–S5). For example, in the subgroup of younger age, the positive association with mortality of liver cancer and pancreatic cancer was stronger, while the positive association was stronger with breast cancer mortality in the older group. In addition, the association with blood cancer mortality was stronger in the subgroup of BMI <30 kg/m^2^ (*P* for interaction <0.05). Sensitivity analysis showed that the associations were essentially unchanged after excluding participants who died within the first 2 years (Supplementary Table S4) or those with poor self-reported overall health at baseline (Supplementary Table S5).

## 4 Discussion

In this prospective cohort study of the general population, we found a positive linear association between serum CysC concentrations and cancer mortality. The site-specific analysis further revealed the positive association with mortality from lung cancer, blood cancer, brain cancer, esophageal cancer, breast cancer, and liver cancer. Our findings suggest an independent adverse effect of CysC on the risk of cancer mortality.

### 4.1 Total cancer mortality

In line with our results, a cohort study including 4,673 participants from the Cardiovascular Health Study reported that compared with the lowest quartile of serum CysC concentrations, the highest quartile was associated with a 79% increased risk of cancer mortality after adjustment for known risk factors and inflammatory biomarkers (Fried et al., 2005). Another prospective cohort study incorporating 5,371 older men also showed that a 50% higher CysC concentration was associated with a 21% increased risk of cancer death (Emberson et al., 2010). However, a biracial cohort study of 3,075 Black and White ambulatory older patients (70–79 years old) with a follow-up of 6 years failed to replicate the association (Shlipak et al., 2006). Moreover, a prior study including 2,990 participants with normal eGFR reported a positive association between serum CysC concentrations and cancer mortality in univariate analysis, which was attenuated to be non-statistically significant in multivariate analysis (HR comparing extreme deciles of CysC = 2.45, 95% CI: 0.85–7.04) (Wu et al., 2010). Generally, the studies observing no association included small numbers of cancer deaths (<350), which might have insufficient statistical power to detect the association. In the current study, which has the largest sample size to date, we ruled out individuals with renal diseases and kidney dysfunction at baseline and controlled for eGFR and other potential confounders, strongly suggesting an independent positive association of CysC concentrations with cancer mortality.

### 4.2 Site-specific cancer mortality

To the best of our knowledge, there is no epidemiologic evidence about the association between CysC concentrations and cancer-specific mortality in the general population. In support of our findings, previous case–control studies found that compared with healthy controls, elevated circulating levels of CysC were detected in patients diagnosed with lung cancer (Chen et al., 2011), esophageal cancer (Yan et al., 2017), breast cancer (Decock et al., 2008), and liver disease (Zinkin et al., 2008). In addition, several retrospective studies conducted in cancer patients have assessed the clinical prognosis significance of CysC. For example, a prior study enrolling 205 patients with small-cell lung cancer found that higher levels of serum CysC were associated with a poorer progression-free survival (Wang H. et al., 2021), and other studies reported CysC as a possible useful biomarker in clinical prognosis management of patients with breast cancer (Leto and Sepporta, 2020), non-Hodgkin B-cell lymphoma (Mulaomerovic et al., 2007), and multiple myeloma (Terpos et al., 2009).

Experimental investigations suggest that CysC plays a critical role in key events of carcinogenesis, such as cell proliferation, apoptosis, and cell adhesion, through its inhibiting activity on cysteine proteases or other cathepsin inhibition-independent mechanisms (Breznik et al., 2019). For example, cysteine proteases have shown the ability to mediate programmed cell death of lung and blood cancers (Broker et al., 2004; Sukhai et al., 2013) and to promote the maturation of antigen-presenting cells, antigen processing, and presentation to T cells (Olson and Joyce, 2015). By thwarting the effects of cysteine proteases, CysC could facilitate cancer cell growth (Leto et al., 2018) and impair T-cell-dependent-antitumor immune response (Zavasnik-Bergant et al., 2005; Magister and Kos, 2013). On the other hand, *ex vivo* and *in vitro* studies have shown a reduction in the proliferation of tumor cells with CysC knockout, indicating that CysC might directly regulate tumor growth through the p38 MAPK signaling pathway. (Završnik et al., 2017). Additional evidence shows that CysC secreted by lung cancer cells could increase the adhesion of cancer cells to the brain microvascular endothelium and result in the formation of brain metastasis (Rai et al., 2015). Moreover, CysC may be conducive to tumor cell invasion and angiogenesis by protecting matrix metalloproteinase-9 from autolysis (Mira et al., 2004; Paupert et al., 2008). Zhao et al. also reported that elevated expression of CST3, the gene encoding cystatin C, was critical for cellular polyploidization that may facilitate cancer cells to resist radiation therapy (Zhao et al., 2023).

The main strengths of this research include the large sample size, prospective design with a long-term follow-up, and accurate assessment of cancer death. Our results were robust to extensive statistical adjustments and sensitivity analyses. Nevertheless, several limitations should be addressed. First, the current study is observational and could not rule out the possibility of residual confounding. Second, a single measurement of serum CysC at baseline was used in the study, which did not take into account the change of the biomarker during the follow-up time. Third, because most of the participants in the UK Biobank were of White ethnicity, the generalization of our findings to other ethnicities should be interpreted with caution. Further independent validation is important for causal inference and would ensure that the results can be generalized to a broader population.

## 5 Conclusion

Our results suggest that serum CysC concentrations are positively associated with mortality from total and certain types of cancer in the general healthy population. Future studies are warranted to clarify the underlying mechanisms of CysC in carcinogenesis and uncover the potential of CysC as a target for cancer treatment.

## Data Availability

The original contributions presented in the study are included in the article/Supplementary Material; further inquiries can be directed to the corresponding authors.
