# Supplementary material for: Associations of serum cystatin C concentrations with total mortality and mortality of 12 site-specific cancers
Source: Front Mol Biosci. 2024 Apr 25;11:1209349. doi: 10.3389/fmolb.2024.1209349 (PMC11079135; doi:10.3389/fmolb.2024.1209349)
Supplement: Supplementary file 1 [file DataSheet1.docx]

**Supplementary Tables and Figures:**

**Supplementary Table 1.** Deaths and ICD-10 diagnosis codes for total and site-specific cancers

**Supplementary Table 2.** Baseline characteristics of UK biobank participants with normal creatinine-based eGFR by status of cancer death

**Supplementary Table 3.** Associations of serum cystatin C concentrations with total and 12 site-specific cancer mortality

**Supplementary Table 4.** Associations of serum cystatin C concentrations with total and 12 site-specific cancer mortality after excluding 694 participants who died in the first two years

**Supplementary Table 5.** Associations of serum cystatin C concentrations with total and 12 site-specific cancer mortality after excluding 9604 participants with poor self-reported overall health

**Supplementary Figure 1.** Dose-response association between serum cystatin C concentrations and mortality from lung cancer (A), blood cancer (B), brain cancer (C), esophageal cancer (D), breast cancer (E), and liver cancer (F).

**Supplementary Figure 2.** Forest plots of stratified analyses of the associations between serum cystatin C concentrations and cancer-specific mortality according to age.

**Supplementary Figure 3.** Forest plots of stratified analyses of the associations between serum cystatin C concentrations and cancer-specific mortality according to sex.

**Supplementary Figure 4.** Forest plots of stratified analyses of the associations between serum cystatin C concentrations and cancer-specific mortality according to BMI.

**Supplementary Figure 5.** Forest plots of stratified analyses of the associations between serum cystatin C concentrations and cancer-specific mortality according to smoking status.

| **Supplementary Table 1.** Deaths and ICD-10 diagnosis codes for total and site-specific cancers | | |
| --- | --- | --- |
| Causes of death | ICD-10 code | Number |
| Total cancer | C00-D48 | 5744 |
| Lung cancer | C34 | 1129 |
| Colorectal cancer | C18-C20 | 588 |
| Pancreatic cancer | C25 | 495 |
| Blood cancer | C81-C96 | 420 |
| Brain cancer | C71 | 315 |
| Esophageal cancer | C15 | 280 |
| Breast cancer | C50 | 275 |
| Prostate cancer | C61 | 256 |
| Liver cancer | C22 | 243 |
| Ovarian cancer | C56 | 228 |
| Stomach cancer | C16 | 144 |
| Kidney cancer | C64 | 139 |
| Abbreviation: ICD-10, international classification of diseases 10^th^ revision. | | |

| **Supplementary Table 2.** Baseline characteristics of UK biobank participants with normal creatinine-based eGFR by status of cancer death^a^ | | | |
| --- | --- | --- | --- |
| Characteristic | All | Cancer death status | |
|  |  | Alive | Dead |
| Participants, No. | 241008 | 235264 | 5744 |
| Age at assessment, year | 54.06 (7.93) | 53.93 (7.91) | 59.38(6.74) |
| Female, % | 54 | 54 | 46 |
| White race, % | 93 | 93 | 96 |
| College or university degree, % | 35 | 35 | 28 |
| On fasting when blood draw, % | 5 | 5 | 6 |
| Townsend deprivation index | -1.2 (3.1) | -1.20 (3.13) | -0.84 (3.34) |
| Body mass index, kg/m^2^ | 27.11 (4.85) | 27.10 (4.84) | 27.59 (5.12) |
| Physical activity, MET hour/week | 39.22 (31.96) | 39.23 (31.97) | 38.78 (31.78) |
| Smoking status, %^b^ |  |  |  |
| Never | 55 | 56 | 37 |
| Previous | 32 | 32 | 39 |
| Current | 12 | 12 | 23 |
| Alcohol consumption, %^b^ |  |  |  |
| Daily or almost daily | 20 | 20 | 26 |
| Three or four times a week | 24 | 24 | 21 |
| Once or twice a week | 26 | 26 | 22 |
| One to three times a month | 11 | 11 | 10 |
| Special occasions only | 11 | 11 | 12 |
| Never | 8 | 8 | 9 |
| Prevalent hypertension, % | 23 | 23 | 33 |
| Prevalent diabetes, % | 5 | 5 | 9 |
| Prevalent CVD, % | 4 | 4 | 8 |
| Postmenopausal, %^c^ | 28 | 28 | 33 |
| Ever HRT use, %^c^ | 17 | 17 | 21 |
| Family history of cancer, % | 34 | 34 | 39 |
| CysC, mg/L | 0.85 (0.11) | 0.85 (0.11) | 0.90 (0.13) |
| eGFR, mL/min/1.73 m^2^ | 99.91 (6.73) | 99.96 (6.74) | 97.80 (6.01) |
| C-reactive protein, mg/L | 2.45 (4.17) | 2.43 (4.14) | 3.33 (5.32) |
| Cholesterol, nmol/L | 5.70 (1.12) | 5.70 (1.11) | 5.60 (1.20) |
| Low-density lipoprotein, nmol/L | 3.56 (0.85) | 3.57 (0.85) | 3.49 (0.90) |
| ^a^ Normal creatinine-based eGFR was defined by CKD-EPI equation ≥ 90 ml/min/1.73 m^2^. Values are means (SD) unless otherwise indicated. | | | |
| ^b^ The total did not sum to 100% because small proportions of participants chose "prefer not to answer". | | | |
| ^c^ Among females only. | | | |
| Abbreviations: eGFR, estimated glomerular filtration rate; CysC, cystatin C; MET, metabolic equivalent task; CVD, cardiovascular disease; HRT, hormone replacement therapy CKD-EPI, Chronic Kidney Disease Epidemiology Collaboration; SD, standard deviation. | | | |

| **Supplementary Table 3.** Associations of serum cystatin C concentrations with total and 12 site-specific cancer mortality | | | | | | | |
| --- | --- | --- | --- | --- | --- | --- | --- |
|  | Quintiles of CysC, mg/L | | | | | *P* for trend | HR per 1-SD increment |
|  | Q1 (0.36-0.76) | Q2 (0.76-0.82) | Q3 (0.82-0.87) | Q4 (0.87-0.94) | Q5 (0.94-4.19) |  |  |
| No. at risk | 48123 | 48491 | 47674 | 48378 | 48342 |  |  |
| All cancer deaths |  |  |  |  |  |  |  |
| No. of event | 705 | 865 | 1000 | 1278 | 1896 |  |  |
| Model 1, HR (95% CI)^a^ | ref | 0.98 (0.88-1.08) | 1.02 (0.93-1.13) | 1.16 (1.06-1.28) | 1.57 (1.43-1.71) | <0.0001 | 1.24 (1.21-1.27) |
| Model 2, HR (95% CI)^b^ | ref | 0.96 (0.87-1.06) | 0.98 (0.89-1.09) | 1.07 (0.97-1.17) | 1.24 (1.13-1.37) | <0.0001 | 1.12 (1.09-1.16) |
| Model 3, HR (95% CI)^c^ | ref | 0.96 (0.83-1.10) | 1.08 (0.95-1.23) | 1.16 (1.01-1.32) | 1.35 (1.18-1.54) | <0.0001 | 1.16 (1.12-1.20) |
| Lung cancer deaths |  |  |  |  |  |  |  |
| No. of event | 113 | 147 | 179 | 247 | 443 |  |  |
| Model 1, HR (95% CI)^a^ | ref | 1.00 (0.78-1.27) | 1.08 (0.85-1.37) | 1.30 (1.04-1.64) | 2.07 (1.67-2.57) | <0.0001 | 1.40 (1.32-1.48) |
| Model 2, HR (95% CI)^b^ | ref | 0.90 (0.70-1.15) | 0.91 (0.72-1.16) | 0.98 (0.78-1.23) | 1.08 (0.86-1.36) | 0.02 | 1.08 (1.01-1.15) |
| Model 3, HR (95% CI)^c^ | ref | 0.94 (0.74-1.21) | 0.99 (0.78-1.26) | 1.08 (0.85-1.37) | 1.22 (0.97-1.55) | 0.001 | 1.12 (1.05-1.20) |
| Colorectal cancer deaths | |  |  |  |  |  |  |
| No. of event | 84 | 100 | 95 | 145 | 164 |  |  |
| Model 1, HR (95% CI)^a^ | ref | 0.95 (0.71-1.28) | 0.82 (0.61-1.11) | 1.12 (0.85-1.48) | 1.05 (0.73-1.52) | 0.20 | 1.06 (0.97-1.16) |
| Model 2, HR (95% CI)^b^ | ref | 0.96 (0.71-1.28) | 0.82 (0.61-1.11) | 1.12 (0.84-1.49) | 1.12 (0.84-1.50) | 0.46 | 1.04 (0.94-1.14) |
| Model 3, HR (95% CI)^c^ | ref | 1.00 (0.74-1.34) | 0.88 (0.64-1.19) | 1.21 (0.90-1.62) | 1.23 (0.90-1.67) | 0.22 | 1.06 (0.96-1.17) |
| Pancreatic cancer deaths | |  |  |  |  |  |  |
| No. of event | 62 | 85 | 90 | 114 | 144 |  |  |
| Model 1, HR (95% CI)^a^ | ref | 1.08 (0.77-1.50) | 1.03 (0.74-1.42) | 1.15 (0.84-1.58) | 1.31 (0.96-1.79) | 0.03 | 1.11 (1.01-1.22) |
| Model 2, HR (95% CI)^b^ | ref | 1.03 (0.74-1.43) | 0.95 (0.68-1.32) | 1.01 (0.73-1.40) | 1.02 (0.74-1.41) | 0.95 | 1.00 (0.91-1.11) |
| Model 3, HR (95% CI)^c^ | ref | 1.07 (0.77-1.49) | 1.01 (0.72-1.41) | 1.09 (0.78-1.52) | 1.12 (0.80-1.58) | 0.50 | 1.04 (0.93-1.16) |
| Blood cancer deaths | |  |  |  |  |  |  |
| No. of event | 48 | 52 | 75 | 93 | 152 |  |  |
| Model 1, HR (95% CI)^a^ | ref | 0.82 (0.56-1.22) | 1.05 (0.73-1.52) | 1.14 (0.80-1.63) | 1.66 (1.19-2.33) | <0.0001 | 1.31 (1.19-1.45) |
| Model 2, HR (95% CI)^b^ | ref | 0.82 (0.55-1.22) | 1.04 (0.72-1.50) | 1.10 (0.77-1.59) | 1.53 (1.07-2.19) | <0.0001 | 1.28 (1.15-1.43) |
| Model 3, HR (95% CI)^c^ | ref | 0.85 (0.57-1.26) | 1.09 (0.74-1.58) | 1.16 (0.80-1.68) | 1.60 (1.10-2.33) | <0.0001 | 1.29 (1.16-1.44) |
| Brain cancer deaths |  |  |  |  |  |  |  |
| No. of event | 43 | 55 | 60 | 71 | 86 |  |  |
| Model 1, HR (95% CI)^a^ | ref | 1.08 (0.72-1.61) | 1.10 (0.74-1.64) | 1.19 (0.81-1.76) | 1.35 (0.92-1.99) | 0.01 | 1.17 (1.04-1.31) |
| Model 2, HR (95% CI)^b^ | ref | 1.04 (0.69-1.55) | 1.04 (0.70-1.55) | 1.13 (0.76-1.68) | 1.31 (0.88-1.96) | 0.02 | 1.17 (1.03-1.33) |
| Model 3, HR (95% CI)^c^ | ref | 1.04 (0.69-1.56) | 1.05 (0.70-1.58) | 1.14 (0.76-1.72) | 1.34 (0.88-2.04) | 0.01 | 1.19 (1.04-1.36) |
| Esophageal cancer deaths | |  |  |  |  |  |  |
| No. of event | 28 | 36 | 52 | 58 | 106 |  |  |
| Model 1, HR (95% CI)^a^ | ref | 0.89 (0.54-1.47) | 1.09 (0.68-1.73) | 1.02 (0.65-1.62) | 1.62 (1.05-2.49) | <0.0001 | 1.28 (1.14-1.45) |
| Model 2, HR (95% CI)^b^ | ref | 0.85 (0.52-1.40) | 1.00 (0.63-1.59) | 0.88 (0.55-1.41) | 1.17 (0.75-1.84) | 0.09 | 1.12 (0.98-1.28) |
| Model 3, HR (95% CI)^c^ | ref | 0.93 (0.56-1.53) | 1.14 (0.71-1.83) | 1.05 (0.65-1.69) | 1.46 (0.91-2.33) | 0.01 | 1.20 (1.05-1.37) |
| Breast cancer deaths | |  |  |  |  |  |  |
| No. of event | 64 | 56 | 52 | 45 | 58 |  |  |
| Model 1, HR (95% CI)^a^ | ref | 1.03 (0.72-1.48) | 1.13 (0.78-1.63) | 1.09 (0.74-1.61) | 1.64 (1.13-2.37) | 0.002 | 1.22 (1.08-1.38) |
| Model 2, HR (95% CI)^b^ | ref | 1.04 (0.72-1.49) | 1.13 (0.73-1.64) | 1.07 (0.71-1.60) | 1.51 (1.01-2.28) | 0.01 | 1.20 (1.04-1.37) |
| Model 3, HR (95% CI)^c^ | ref | 1.03 (0.71-1.48) | 1.11 (0.75-1.63) | 1.04 (0.69-1.53) | 1.44 (0.94-2.21) | 0.02 | 1.18 (1.03-1.36) |
| Prostate cancer deaths | |  |  |  |  |  |  |
| No. of event | 18 | 33 | 50 | 66 | 89 |  |  |
| Model 1, HR (95% CI)^a^ | ref | 0.95 (0.53-1.68) | 1.05 (0.61-1.80) | 1.07 (0.63-1.80) | 1.14 (0.69-1.90) | 0.42 | 1.06 (0.92-1.21) |
| Model 2, HR (95% CI)^b^ | ref | 0.95 (0.53-1.68) | 1.05 (0.62-1.81) | 1.04 (0.61-1.75) | 1.07 (0.63-1.80) | 0.77 | 1.02 (0.89-1.18) |
| Model 3, HR (95% CI)^c^ | ref | 0.97 (0.54-1.72) | 1.09 (0.63-1.88) | 1.09 (0.63-1.86) | 1.13 (0.66-1.94) | 0.62 | 1.04 (0.89-1.21) |
| Liver cancer deaths |  |  |  |  |  |  |  |
| No. of event | 24 | 35 | 32 | 47 | 105 |  |  |
| Model 1, HR (95% CI)^a^ | ref | 1.12(0.67-1.89) | 0.91 (0.53-1.56) | 1.17 (0.71-1.94) | 2.32 (1.46-3.68) | <0.0001 | 1.50 (1.33-1.68) |
| Model 2, HR (95% CI)^b^ | ref | 1.11 (0.66-1.87) | 0.89 (0.52-1.52) | 1.09 (0.65-1.81) | 1.89 (1.16-3.06) | <0.0001 | 1.38 (1.21-1.58) |
| Model 3, HR (95% CI)^c^ | ref | 1.25 (0.74-2.13) | 1.06 (0.61-1.83) | 1.38 (0.81-2.33) | 2.52 (1.52-4.18) | <0.0001 | 1.49 (1.31-1.69) |
| Ovarian cancer deaths | |  |  |  |  |  |  |
| No. of event | 48 | 48 | 53 | 39 | 40 |  |  |
| Model 1, HR (95% CI)^a^ | ref | 1.01 (0.67-1.51) | 1.19 (0.80-1.77) | 0.91 (0.59-1.40) | 1.01 (0.65-1.55) | 0.81 | 0.98 (0.86-1.13) |
| Model 2, HR (95% CI)^b^ | ref | 0.99 (0.66-1.48) | 1.15 (0.77-1.72) | 0.85 (0.54-1.32) | 0.88 (0.55-1.40) | 0.39 | 0.94 (0.81-1.09) |
| Model 3, HR (95% CI)^c^ | ref | 1.03 (0.68-1.54) | 1.21 (0.80-1.82) | 0.90 (0.57-1.43) | 0.95 (0.58-1.54) | 0.61 | 0.96 (0.82-1.12) |
| Stomach cancer deaths | |  |  |  |  |  |  |
| No. of event | 21 | 22 | 24 | 30 | 47 |  |  |
| Model 1, HR (95% CI)^a^ | ref | 0.78 (0.43-1.43) | 0.75 (0.41-1.35) | 0.81 (0.46-1.44) | 1.13 (0.66-1.94) | 0.10 | 1.16 (0.97-1.38) |
| Model 2, HR (95% CI)^b^ | ref | 0.74 (0.40-1.35) | 0.66 (0.36-1.21) | 0.66 (0.37-1.19) | 0.74 (0.41-1.31) | 0.82 | 0.98 (0.81-1.18) |
| Model 3, HR (95% CI)^c^ | ref | 0.76 (0.41-1.39) | 0.69 (0.37-1.27) | 0.70 (0.38-1.28) | 0.78 (0.42-1.43) | 0.99 | 1.00 (0.82-1.22) |
| Kidney cancer deaths | |  |  |  |  |  |  |
| No. of event | 15 | 23 | 26 | 27 | 48 |  |  |
| Model 1, HR (95% CI)^a^ | ref | 1.09 (0.57-2.09) | 1.05 (0.55-1.99) | 0.93 (0.49-1.76) | 1.44 (0.79-2.63) | 0.17 | 1.13 (0.95-1.36) |
| Model 2, HR (95% CI)^b^ | ref | 1.03 (0.53-1.97) | 0.95 (0.50-1.81) | 0.79 (0.41-1.51) | 1.05 (0.56-1.96) | 0.94 | 0.99 (0.82-1.20) |
| Model 3, HR (95% CI)^c^ | ref | 1.02 (0.53-1.97) | 0.95 (0.49-1.83) | 0.78 (0.40-1.52) | 1.00 (0.52-1.92) | 0.11 | 0.97 (0.79-1.18) |
| ^a^ Cox proportional hazards models were used to calculate hazard ratios (HR) and 95% confidence intervals (CI) for cancer mortality according to quintiles of CysC concentrations. Model 1: adjusted for age at blood draw (years), sex (female, male), ethnicity (white, not white), and fasting status (yes, no). | | | | | | | |
| ^b^ Model 2: additionally adjusted for Townsend deprivation index (continuous), college or university degree (yes, no), body mass index (kg/m^2^), smoking status (never, previous, current), pack years of smoking (continuous), alcohol consumption (never, special occasions only, 1-3 times per month, 1-2 times per week, 3-4 times per week, daily/almost daily), physical activity (MET-hours/week), family history of cancer (yes, no), prevalent hypertension (yes, no), prevalent diabetes (yes, no), prevalent cardiovascular diseases (yes, no), and for women, menopause status (yes, no) and hormone replacement therapy (yes, no). | | | | | | | |
| ^c^ Model 3: additionally adjusted for eGFR (mL/min/1.73 m^2^), C-reactive protein (mg/L), cholesterol (mmol/L), and low-density lipoprotein (mmol/L). | | | | | | | |
| Abbreviations: CysC, cystatin C; HR, hazard ratio; SD, standard deviation; CI, confidence interval; ref, reference; MET, metabolic equivalent task. | | | | | | | |

| **Supplementary Table 4.** Associations of serum cystatin C concentrations with total and 12 site-specific cancer mortality after excluding 694 participants who died in the first two years | | | | | | | |
| --- | --- | --- | --- | --- | --- | --- | --- |
|  | Quintiles of CysC, mg/L | | | | | *P* for trend | HR per log SD increment |
|  | Q1 (0.36-0.76) | Q2 (0.76-0.82) | Q3 (0.82-0.87) | Q4 (0.87-0.94) | Q5 (0.94-4.19) |  |  |
| No. at risk | 48057 | 48401 | 47561 | 48238 | 48057 |  |  |
| All cancer deaths |  |  |  |  |  |  |  |
| No. of event | 679 | 830 | 947 | 1203 | 1799 |  |  |
| Model 1, HR (95% CI)^a^ | ref | 0.97 (0.88-1.08) | 1.01 (0.91-1.11) | 1.14 (1.04-1.26) | 1.56 (1.42-1.71) | <0.0001 | 1.24 (1.20-1.27) |
| Model 2, HR (95% CI)^b^ | ref | 0.96 (0.87-1.06) | 0.97 (0.88-1.07) | 1.05 (0.95-1.16) | 1.23 (1.12-1.36) | <0.0001 | 1.12 (1.09-1.16) |
| Model 3, HR (95% CI)^c^ | ref | 1.00 (0.90-1.11) | 1.04 (0.94-1.15) | 1.14 (1.03-1.26) | 1.36 (1.23-1.51) | <0.0001 | 1.16 (1.12-1.20) |
| Lung cancer deaths |  |  |  |  |  |  |  |
| No. of event | 104 | 139 | 166 | 232 | 418 |  |  |
| Model 1, HR (95% CI)^a^ | ref | 1.03 (0.80-1.33) | 1.09 (0.85-1.40) | 1.34 (1.06-1.70) | 2.16 (1.73-2.70) | <0.0001 | 1.41 (1.34-1.51) |
| Model 2, HR (95% CI)^b^ | ref | 0.93 (0.72-1.20) | 0.92 (0.72-1.19) | 1.00 (0.79-1.28) | 1.12 (0.89-1.42) | 0.01 | 1.10 (1.03-1.17) |
| Model 3, HR (95% CI)^c^ | ref | 0.97 (0.75-1.26) | 1.00 (0.78-1.29) | 1.11 (0.87-1.42) | 1.27 (0.99-1.62) | 0.0002 | 1.14 (1.07-1.22) |
| Colorectal cancer deaths | |  |  |  |  |  |  |
| No. of event | 83 | 97 | 87 | 138 | 157 |  |  |
| Model 1, HR (95% CI)^a^ | ref | 0.94 (0.70-1.26) | 0.77 (0.57-1.04) | 1.09 (0.83-1.45) | 1.14 (0.86-1.51) | 0.22 | 1.06 (0.97-1.16) |
| Model 2, HR (95% CI)^b^ | ref | 0.94 (0.70-1.27) | 0.77 (0.57-1.05) | 1.09 (0.82-1.46) | 1.11 (0.83-1.50) | 0.43 | 1.04 (0.94-1.14) |
| Model 3, HR (95% CI)^c^ | ref | 0.98 (0.73-1.33) | 0.82 (0.60-1.12) | 1.19 (0.88-1.60) | 1.23 (0.90-1.68) | 0.17 | 1.07 (0.97-1.19) |
| Pancreatic cancer deaths | |  |  |  |  |  |  |
| No. of event | 60 | 78 | 84 | 104 | 137 |  |  |
| Model 1, HR (95% CI)^a^ | ref | 1.02 (0.73-1.44) | 0.99 (0.71-1.39) | 1.09 (0.79-1.51) | 1.30 (0.95-1.79) | 0.03 | 1.11 (1.01-1.23) |
| Model 2, HR (95% CI)^b^ | ref | 0.98 (0.70-1.38) | 0.92 (0.66-1.29) | 0.96 (0.69-1.33) | 1.01 (0.72-1.41) | 0.92 | 1.01 (0.91-1.12) |
| Model 3, HR (95% CI)^c^ | ref | 1.01 (0.72-1.42) | 0.96 (0.68-1.36) | 1.02 (0.72-1.43) | 1.10 (0.77-1.56) | 0.53 | 1.04 (0.93-1.16) |
| Blood cancer deaths | |  |  |  |  |  |  |
| No. of event | 47 | 52 | 68 | 91 | 139 |  |  |
| Model 1, HR (95% CI)^a^ | ref | 0.84 (0.57-1.25) | 0.98 (0.67-1.42) | 1.14 (0.80-1.64) | 1.56 (1.11-2.20) | <0.0001 | 1.28 (1.15-1.42) |
| Model 2, HR (95% CI)^b^ | ref | 0.84 (0.57-1.26) | 0.97 (0.66-1.42) | 1.11 (0.77-1.61) | 1.47 (1.02-2.11) | <0.0001 | 1.25 (1.12-1.40) |
| Model 3, HR (95% CI)^c^ | ref | 0.87 (0.58-1.30) | 1.01 (0.69-1.49) | 1.17 (0.80-1.72) | 1.54 (1.05-2.26) | <0.0001 | 1.27 (1.13-1.42) |
| Brain cancer deaths |  |  |  |  |  |  |  |
| No. of event | 41 | 52 | 57 | 63 | 79 |  |  |
| Model 1, HR (95% CI)^a^ | ref | 1.08 (0.71-1.63) | 1.11 (0.74-1.67) | 1.12 (0.75-1.69) | 1.33 (0.89-1.97) | 0.03 | 1.15 (1.02-1.30) |
| Model 2, HR (95% CI)^b^ | ref | 1.03 (0.68-1.56) | 1.04 (0.69-1.57) | 1.05 (0.70-1.59) | 1.26 (0.83-1.91) | 0.05 | 1.14 (1.00-1.30) |
| Model 3, HR (95% CI)^c^ | ref | 1.04 (0.69-1.58) | 1.06 (0.69-1.60) | 1.08 (0.71-1.65) | 1.31 (0.85-2.02) | 0.01 | 1.17 (1.02-1.34) |
| Esophageal cancer deaths | |  |  |  |  |  |  |
| No. of event | 27 | 34 | 52 | 53 | 100 |  |  |
| Model 1, HR (95% CI)^a^ | ref | 0.88 (0.53-1.46) | 1.13 (0.71-1.81) | 0.98 (0.61-1.57) | 1.60 (1.03-2.48) | 0.0003 | 1.27 (1.12-1.44) |
| Model 2, HR (95% CI)^b^ | ref | 0.83 (0.50-1.38) | 1.04 (0.65-1.66) | 0.83 (0.52-1.34) | 1.13 (0.71-1.79) | 0.20 | 1.09 (0.96-1.25) |
| Model 3, HR (95% CI)^c^ | ref | 0.90 (0.54-1.50) | 1.16 (0.72-1.88) | 0.97 (0.60-1.59) | 1.37 (0.85-2.22) | 0.03 | 1.17 (1.01-1.34) |
| Breast cancer deaths | |  |  |  |  |  |  |
| No. of event | 64 | 55 | 52 | 43 | 53 |  |  |
| Model 1, HR (95% CI)^a^ | ref | 1.01 (0.70-1.45) | 1.12 (0.77-1.63) | 1.04 (0.70-1.54) | 1.49 (1.02-2.17) | 0.02 | 1.17 (1.03-1.32) |
| Model 2, HR (95% CI)^b^ | ref | 1.02 (0.71-1.46) | 1.12 (0.77-1.63) | 1.01 (0.67-1.53) | 1.38 (0.91-2.09) | 0.06 | 1.14 (1.00-1.31) |
| Model 3, HR (95% CI)^c^ | ref | 1.00 (0.69-1.45) | 1.10 (0.75-1.61) | 0.98 (0.64-1.50) | 1.30 (0.84-2.01) | 0.11 | 1.12 (0.97-1.30) |
| Prostate cancer deaths | |  |  |  |  |  |  |
| No. of event | 18 | 33 | 50 | 63 | 87 |  |  |
| Model 1, HR (95% CI)^a^ | ref | 0.95 (0.53-1.68) | 1.05 (0.62-1.81) | 1.02 (0.60-1.72) | 1.12 (0.67-1.86) | 0.53 | 1.05 (0.91-1.20) |
| Model 2, HR (95% CI)^b^ | ref | 0.95 (0.53-1.68) | 1.05 (0.61-1.81) | 1.00 (0.59-1.70) | 1.06 (0.63-1.79) | 0.80 | 1.02 (0.88-1.18) |
| Model 3, HR (95% CI)^c^ | ref | 0.97 (0.55-1.74) | 1.10 (0.64-1.90) | 1.06 (0.62-1.81) | 1.15 (0.67-1.97) | 0.59 | 1.04 (0.90-1.21) |
| Liver cancer deaths |  |  |  |  |  |  |  |
| No. of event | 22 | 32 | 31 | 44 | 98 |  |  |
| Model 1, HR (95% CI)^a^ | ref | 1.11 (0.64-1.92) | 0.95 (0.55-1.65) | 1.18 (0.70-1.99) | 2.32 (1.44-3.75) | <0.0001 | 1.50 (1.33-1.69) |
| Model 2, HR (95% CI)^b^ | ref | 1.09 (0.63-1.89) | 0.92 (0.53-1.60) | 1.08 (0.64-1.84) | 1.86 (1.12-3.07) | <0.0001 | 1.38 (1.20-1.58) |
| Model 3, HR (95% CI)^c^ | ref | 1.23 (0.71-2.14) | 1.09 (0.62-1.92) | 1.36 (0.79-2.35) | 2.46 (1.45-4.17) | <0.0001 | 1.48 (1.30-1.70) |
| Ovarian cancer deaths | |  |  |  |  |  |  |
| No. of event | 46 | 47 | 51 | 37 | 40 |  |  |
| Model 1, HR (95% CI)^a^ | ref | 1.03 (0.68-1.55) | 1.20 (0.80-1.79) | 0.90 (0.58-1.40) | 1.05 (0.68-1.63) | 0.97 | 1.00 (0.87-1.15) |
| Model 2, HR (95% CI)^b^ | ref | 1.01 (0.67-1.52) | 1.15 (0.76-1.73) | 0.84 (0.53-1.32) | 0.92 (0.57-1.47) | 0.50 | 0.95 (0.82-1.11) |
| Model 3, HR (95% CI)^c^ | ref | 1.06 (0.70-1.60) | 1.22 (0.80-1.86) | 0.91 (0.57-1.45) | 1.01 (0.61-1.65) | 0.82 | 0.98 (0.84-1.15) |
| Stomach cancer deaths | |  |  |  |  |  |  |
| No. of event | 17 | 20 | 23 | 29 | 43 |  |  |
| Model 1, HR (95% CI)^a^ | ref | 0.89 (0.46-1.71) | 0.90 (0.48-1.70) | 0.99 (0.54-1.83) | 1.31 (0.73-2.36) | 0.05 | 1.20 (1.00-1.44) |
| Model 2, HR (95% CI)^b^ | ref | 0.82 (0.43-1.57) | 0.77 (0.40-1.46) | 0.77 (0.40-1.44) | 0.80 (0.43-1.49) | 0.94 | 0.99 (0.82-1.21) |
| Model 3, HR (95% CI)^c^ | ref | 0.83 (0.43-1.60) | 0.78 (0.41-1.50) | 0.79 (0.42-1.51) | 0.82 (0.43-1.58) | 0.94 | 1.01 (0.82-1.24) |
| Kidney cancer deaths | |  |  |  |  |  |  |
| No. of event | 15 | 23 | 25 | 24 | 44 |  |  |
| Model 1, HR (95% CI)^a^ | ref | 1.09 (0.57-2.10) | 1.01 (0.53-1.94) | 0.83 (0.43-1.60) | 1.33 (0.73-2.45) | 0.33 | 1.10 (0.91-1.32) |
| Model 2, HR (95% CI)^b^ | ref | 1.03 (0.53-1.98) | 0.92 (0.48-1.76) | 0.70 (0.36-1.36) | 0.96 (0.51-1.81) | 0.65 | 0.96 (0.79-1.16) |
| Model 3, HR (95% CI)^c^ | ref | 1.03 (0.53-1.98) | 0.92 (0.48-1.79) | 0.70 (0.35-1.39) | 0.93 (0.48-1.81) | 0.55 | 0.94 (0.77-1.15) |
| ^a^ Cox proportional hazards models were used to calculate hazard ratios (HR) and 95% confidence intervals (CI) for cancer mortality according to quintiles of CysC concentrations. Model 1: adjusted for age at blood draw (years), sex (female, male), ethnicity (white, not white), and fasting status (yes, no). | | | | | | | |
| ^b^ Model 2: additionally adjusted for Townsend deprivation index (continuous), college or university degree (yes, no), body mass index (kg/m^2^), smoking status (never, previous, current), pack years of smoking (continuous), alcohol consumption (never, special occasions only, 1-3 times per month, 1-2 times per week, 3-4 times per week, daily/almost daily), physical activity (MET-hours/week), family history of cancer (yes, no), prevalent hypertension (yes, no), prevalent diabetes (yes, no), prevalent cardiovascular diseases (yes, no), and for women, menopause status (yes, no) and hormone replacement therapy (yes, no). | | | | | | | |
| ^c^ Model 3: additionally adjusted for eGFR (mL/min/1.73 m^2^), C-reactive protein (mg/L), cholesterol (mmol/L), and low-density lipoprotein (mmol/L). | | | | | | | |
| Abbreviations: CysC, cystatin C; HR, hazard ratio; SD, standard deviation; CI, confidence interval; ref, reference; MET, metabolic equivalent task. | | | | | | | |

| **Supplementary Table 5.** Associations of serum cystatin C concentrations with total and 12 site-specific cancer mortality after excluding 9604 participants with poor self-reported overall health | | | | | | | |
| --- | --- | --- | --- | --- | --- | --- | --- |
|  | Quintiles of CysC, mg/L | | | | | *P* for trend | HR per log SD increment |
|  | Q1 (0.36-0.75) | Q2 (0.76-0.81) | Q3 (0.81-0.87) | Q4 (0.87-0.94) | Q5 (0.94-4.14) |  |  |
| No. at risk | 46310 | 46045 | 46356 | 46481 | 46212 |  |  |
| All cancer deaths |  |  |  |  |  |  |  |
| No. of event | 668 | 800 | 958 | 1174 | 1739 |  |  |
| Model 1, HR (95% CI)^a^ | ref | 0.96 (0.87-1.07) | 1.02 (0.92-1.13) | 1.12 (1.02-1.24) | 1.51 (1.38-1.66) | <0.0001 | 1.22 (1.18-1.25) |
| Model 2, HR (95% CI)^b^ | ref | 0.95 (0.87-1.07) | 0.99 (0.89-1.09) | 1.04 (0.94-1.15) | 1.22 (1.11-1.35) | <0.0001 | 1.11 (1.08-1.15) |
| Model 3, HR (95% CI)^c^ | ref | 1.00 (0.90-1.11) | 1.04 (0.94-1.15) | 1.14 (1.03-1.26) | 1.36 (1.23-1.51) | <0.0001 | 1.15 (1.11-1.19) |
| Lung cancer deaths |  |  |  |  |  |  |  |
| No. of event | 103 | 130 | 161 | 221 | 398 |  |  |
| Model 1, HR (95% CI)^a^ | ref | 0.97 (0.75-1.26) | 1.04 (0.81-1.34) | 1.26 (0.99-1.60) | 2.00 (1.60-2.51) | <0.0001 | 1.39 (1.30-1.48) |
| Model 2, HR (95% CI)^b^ | ref | 0.90 (0.69-1.16) | 0.91 (0.71-1.17) | 0.99 (0.77-1.26) | 1.12 (0.88-1.41) | 0.01 | 1.09 (1.02-1.17) |
| Model 3, HR (95% CI)^c^ | ref | 0.94 (0.72-1.22) | 0.99 (0.77-1.28) | 1.10 (0.86-1.41) | 1.27 (0.99-1.63) | 0.0004 | 1.13 (1.06-1.22) |
| Colorectal cancer deaths | |  |  |  |  |  |  |
| No. of event | 80 | 94 | 96 | 133 | 158 |  |  |
| Model 1, HR (95% CI)^a^ | ref | 0.96 (0.71-1.29) | 0.87 (0.64-1.17) | 1.09 (0.82-1.45) | 1.19 (0.90-1.58) | 0.21 | 1.06 (0.97-1.16) |
| Model 2, HR (95% CI)^b^ | ref | 0.96 (0.71-1.30) | 0.87 (0.64-1.18) | 1.09 (0.81-1.45) | 1.14 (0.85-1.54) | 0.50 | 1.03 (0.94-1.14) |
| Model 3, HR (95% CI)^c^ | ref | 1.01 (0.74-1.37) | 0.94 (0.69-1.28) | 1.20 (0.88-1.62) | 1.28 (0.94-1.75) | 0.18 | 1.07 (0.97-1.18) |
| Pancreatic cancer deaths | |  |  |  |  |  |  |
| No. of event | 60 | 79 | 88 | 111 | 129 |  |  |
| Model 1, HR (95% CI)^a^ | ref | 1.05 (0.75-1.47) | 1.02 (0.73-1.43) | 1.15 (0.84-1.59) | 1.21 (0.88-1.66) | 0.12 | 1.09 (0.98-1.19) |
| Model 2, HR (95% CI)^b^ | ref | 1.00 (0.71-1.40) | 0.95 (0.68-1.32) | 1.01 (0.73-1.41) | 0.95 (0.68-1.32) | 0.77 | 0.99 (0.89-1.09) |
| Model 3, HR (95% CI)^c^ | ref | 1.03 (0.73-1.44) | 0.99 (0.70-1.39) | 1.08 (0.77-1.50) | 1.03 (0.72-1.46) | 0.81 | 1.01 (0.91-1.13) |
| Blood cancer deaths | |  |  |  |  |  |  |
| No. of event | 48 | 46 | 74 | 86 | 144 |  |  |
| Model 1, HR (95% CI)^a^ | ref | 0.74 (0.49-1.11) | 1.03 (0.71-1.48) | 1.05 (0.73-1.51) | 1.57 (1.12-2.20) | <0.0001 | 1.32 (1.19-1.46) |
| Model 2, HR (95% CI)^b^ | ref | 0.74 (0.49-1.12) | 1.03 (0.71-1.49) | 1.04 (0.72-1.50) | 1.50 (1.05-2.15) | <0.0001 | 1.30 (1.17-1.46) |
| Model 3, HR (95% CI)^c^ | ref | 0.76 (0.50-1.14) | 1.06 (0.73-1.55) | 1.08 (0.74-1.58) | 1.54 (1.05-2.24) | <0.0001 | 1.31 (1.17-1.47) |
| Brain cancer deaths |  |  |  |  |  |  |  |
| No. of event | 41 | 56 | 55 | 69 | 84 |  |  |
| Model 1, HR (95% CI)^a^ | ref | 1.16 (0.77-1.74) | 1.04 (0.69-1.56) | 1.20 (0.81-1.79) | 1.37 (0.92-2.03) | 0.02 | 1.16 (1.03-1.30) |
| Model 2, HR (95% CI)^b^ | ref | 1.11 (0.74-1.67) | 0.97 (0.64-1.47) | 1.13 (0.75-1.69) | 1.30 (0.86-1.96) | 0.04 | 1.15 (1.01-1.31) |
| Model 3, HR (95% CI)^c^ | ref | 1.04 (0.69-1.58) | 1.06 (0.69-1.60) | 1.08 (0.71-1.65) | 1.31 (0.85-2.02) | 0.03 | 1.16 (1.02-1.34) |
| Esophageal cancer deaths | |  |  |  |  |  |  |
| No. of event | 25 | 31 | 50 | 54 | 98 |  |  |
| Model 1, HR (95% CI)^a^ | ref | 0.87 (0.51-1.47) | 1.14 (0.70-1.86) | 1.05 (0.65-1.71) | 1.64 (1.04-2.58) | <0.0001 | 1.31 (1.15-1.49) |
| Model 2, HR (95% CI)^b^ | ref | 0.83 (0.49-1.40) | 1.06 (0.65-1.72) | 0.91 (0.56-1.49) | 1.20 (0.75-1.92) | 0.04 | 1.15 (1.01-1.32) |
| Model 3, HR (95% CI)^c^ | ref | 0.90 (0.54-1.50) | 1.16 (0.72-1.88) | 0.97 (0.60-1.59) | 1.37 (0.85-2.22) | 0.01 | 1.22 |
| Breast cancer deaths | |  |  |  |  |  |  |
| No. of event | 65 | 49 | 55 | 44 | 51 |  |  |
| Model 1, HR (95% CI)^a^ | ref | 0.89 (0.61-1.29) | 1.14 (0.79-1.64) | 1.02 (0.69-1.51) | 1.37 (0.93-2.01) | 0.02 | 1.17 (1.03-1.32) |
| Model 2, HR (95% CI)^b^ | ref | 0.90 (0.62-1.31) | 1.15 (0.79-1.67) | 1.01 (0.67-1.52) | 1.30 (0.86-1.98) | 0.04 | 1.16 (1.01-1.33) |
| Model 3, HR (95% CI)^c^ | ref | 0.90 (0.61-1.31) | 1.14 (0.78-1.67) | 1.00 (0.66-1.52) | 1.26 (0.81-1.95) | 0.06 | 1.15 (0.99-1.33) |
| Prostate cancer deaths | |  |  |  |  |  |  |
| No. of event | 18 | 29 | 46 | 60 | 89 |  |  |
| Model 1, HR (95% CI)^a^ | ref | 0.83 (0.46-1.50) | 0.95 (0.55-1.64) | 0.96 (0.57-1.62) | 1.12 (0.67-1.86) | 0.54 | 1.05 (0.91-1.20) |
| Model 2, HR (95% CI)^b^ | ref | 0.84 (0.47-1.51) | 0.95 (0.55-1.65) | 0.94 (0.56-1.61) | 1.08 (0.64-1.83) | 0.79 | 1.02 (0.88-1.18) |
| Model 3, HR (95% CI)^c^ | ref | 0.87 (0.48-1.56) | 1.00 (0.57-1.73) | 1.00 (0.58-1.73) | 1.16 (0.68-2.00) | 0.64 | 1.04 (0.89-1.21) |
| Liver cancer deaths |  |  |  |  |  |  |  |
| No. of event | 20 | 32 | 30 | 39 | 87 |  |  |
| Model 1, HR (95% CI)^a^ | ref | 1.27 (0.72-2.22) | 1.04 (0.59-1.85) | 1.21 (0.70-2.10) | 2.42 (1.46-4.01) | <0.0001 | 1.45 (1.27-1.65) |
| Model 2, HR (95% CI)^b^ | ref | 1.25 (0.71-2.20) | 1.01 (0.57-1.80) | 1.12 (0.64-1.97) | 1.99 (1.17-3.38) | 0.0001 | 1.34 (1.15-1.55) |
| Model 3, HR (95% CI)^c^ | ref | 1.42 (0.80-2.50) | 1.21 (0.67-2.17) | 1.43 (0.81-2.54) | 2.66 (1.53-4.63) | <0.0001 | 1.44 (1.25-1.67) |
| Ovarian cancer deaths | |  |  |  |  |  |  |
| No. of event | 46 | 47 | 48 | 42 | 37 |  |  |
| Model 1, HR (95% CI)^a^ | ref | 1.04 (0.69-1.56) | 1.10 (0.73-1.66) | 1.00 (0.66-1.54) | 0.95 (0.61-1.49) | 0.68 | 0.97 (0.85-1.12) |
| Model 2, HR (95% CI)^b^ | ref | 1.02 (0.67-1.53) | 1.06 (0.70-1.60) | 0.93 (0.60-1.44) | 0.83 (0.51-1.33) | 0.29 | 0.92 (0.79-1.07) |
| Model 3, HR (95% CI)^c^ | ref | 1.06 (0.70-1.60) | 1.12 (0.73-1.71) | 1.00 (0.64-1.58) | 0.90 (0.55-1.49) | 0.53 | 0.95 (0.81-1.11) |
| Stomach cancer deaths | |  |  |  |  |  |  |
| No. of event | 19 | 22 | 22 | 26 | 41 |  |  |
| Model 1, HR (95% CI)^a^ | ref | 0.88 (0.47-1.63) | 0.75 (0.40-1.39) | 0.78 (0.42-1.43) | 1.09 (0.62-1.93) | 0.11 | 1.16 (0.97-1.40) |
| Model 2, HR (95% CI)^b^ | ref | 0.83 (0.45-1.55) | 0.67 (0.36-1.26) | 0.64 (0.35-1.20) | 0.72 (0.39-1.32) | 0.95 | 0.99 (0.82-1.21) |
| Model 3, HR (95% CI)^c^ | ref | 0.85 (0.45-1.59) | 0.69 (0.36-1.31) | 0.67 (0.35-1.27) | 0.76 (0.40-1.44) | 0.81 | 1.03 (0.83-1.27) |
| Kidney cancer deaths | |  |  |  |  |  |  |
| No. of event | 15 | 22 | 25 | 24 | 47 |  |  |
| Model 1, HR (95% CI)^a^ | ref | 1.05 (0.54-2.03) | 0.99 (0.52-1.90) | 0.82 (0.42-1.58) | 1.40 (0.76-2.55) | 0.29 | 1.11 (0.92-1.33) |
| Model 2, HR (95% CI)^b^ | ref | 0.99 (0.51-1.91) | 0.90 (0.47-1.72) | 0.70 (0.36-1.35) | 1.02 (0.54-1.91) | 0.73 | 0.97 (0.80-1.17) |
| Model 3, HR (95% CI)^c^ | ref | 0.98 (0.50-1.91) | 0.90 (0.47-1.75) | 0.70 (0.35-1.38) | 0.98 (0.51-1.90) | 0.59 | 0.95(0.77-1.16) |
| ^a^ Cox proportional hazards models were used to calculate hazard ratios (HR) and 95% confidence intervals (CI) for cancer mortality according to quintiles of CysC concentrations. Model 1: adjusted for age at blood draw (years), sex (female, male), ethnicity (white, not white), and fasting status (yes, no). | | | | | | | |
| ^b^ Model 2: additionally adjusted for Townsend deprivation index (continuous), college or university degree (yes, no), body mass index (kg/m^2^), smoking status (never, previous, current), pack years of smoking (continuous), alcohol consumption (never, special occasions only, 1-3 times per month, 1-2 times per week, 3-4 times per week, daily/almost daily), physical activity (MET-hours/week), family history of cancer (yes, no), prevalent hypertension (yes, no), prevalent diabetes (yes, no), prevalent cardiovascular diseases (yes, no), and for women, menopause status (yes, no) and hormone replacement therapy (yes, no). | | | | | | | |
| ^c^ Model 3: additionally adjusted for eGFR (mL/min/1.73 m^2^), C-reactive protein (mg/L), cholesterol (mmol/L), and low-density lipoprotein (mmol/L). | | | | | | | |
| Abbreviations: CysC, cystatin C; HR, hazard ratio; SD, standard deviation; CI, confidence interval; ref, reference; MET, metabolic equivalent task. | | | | | | | |

**
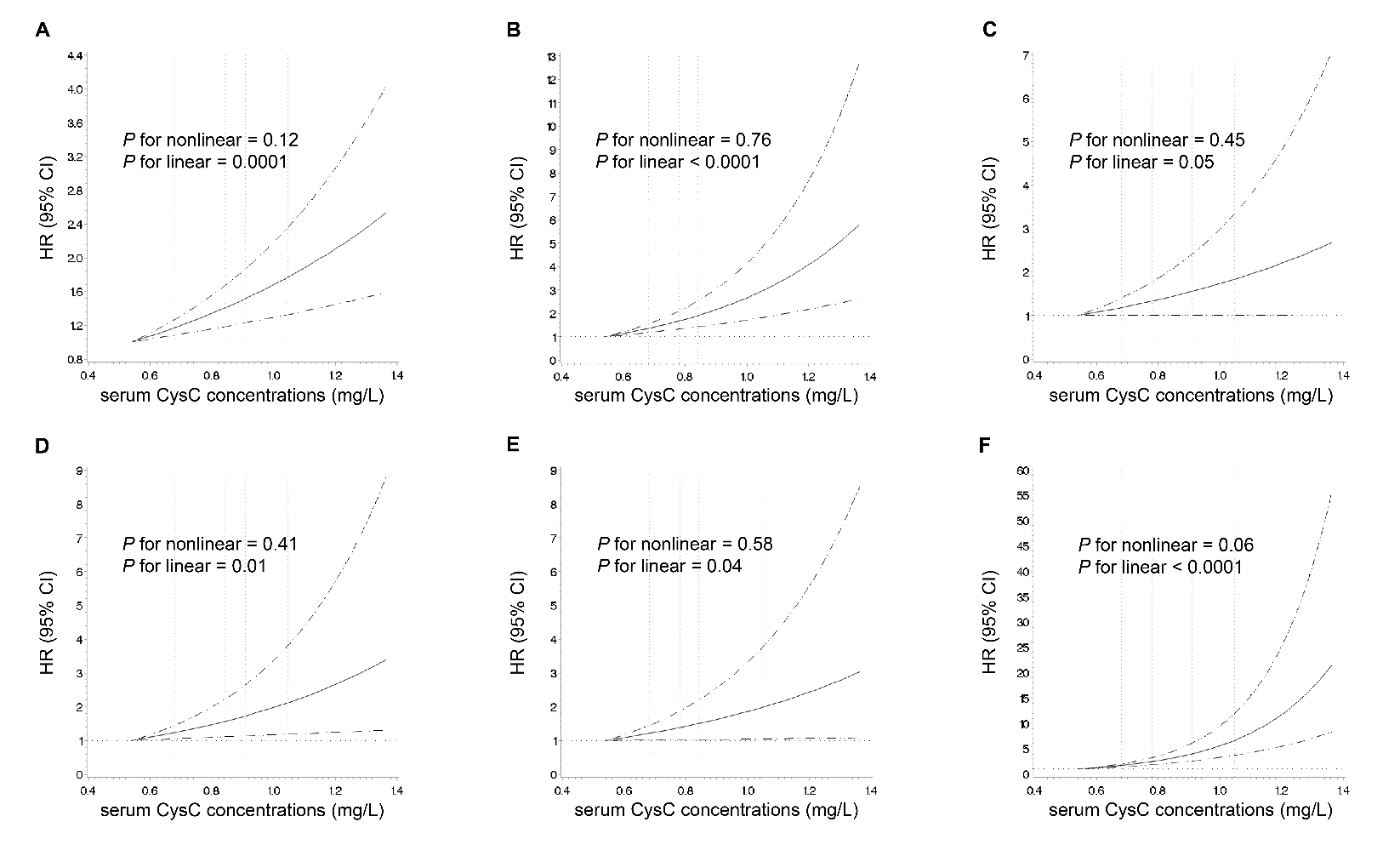
**

**Supplementary Figure 1.** Dose-response association between serum cystatin C concentrations and mortality from lung cancer (A), blood cancer (B), brain cancer (C), esophageal cancer (D), breast cancer (E), and liver cancer (F).

Multivariable Cox regression models with restricted cubic spline analysis were performed, adjusting for the same set of covariates as in Model 3. Cystatin C concentrations above 99.9% and below 0.1% were not plotted due to wide confidence intervals at the extremes. Solid line represents estimates of hazard ratio (HR) and dashed lines represent 95% confidence intervals (CI).


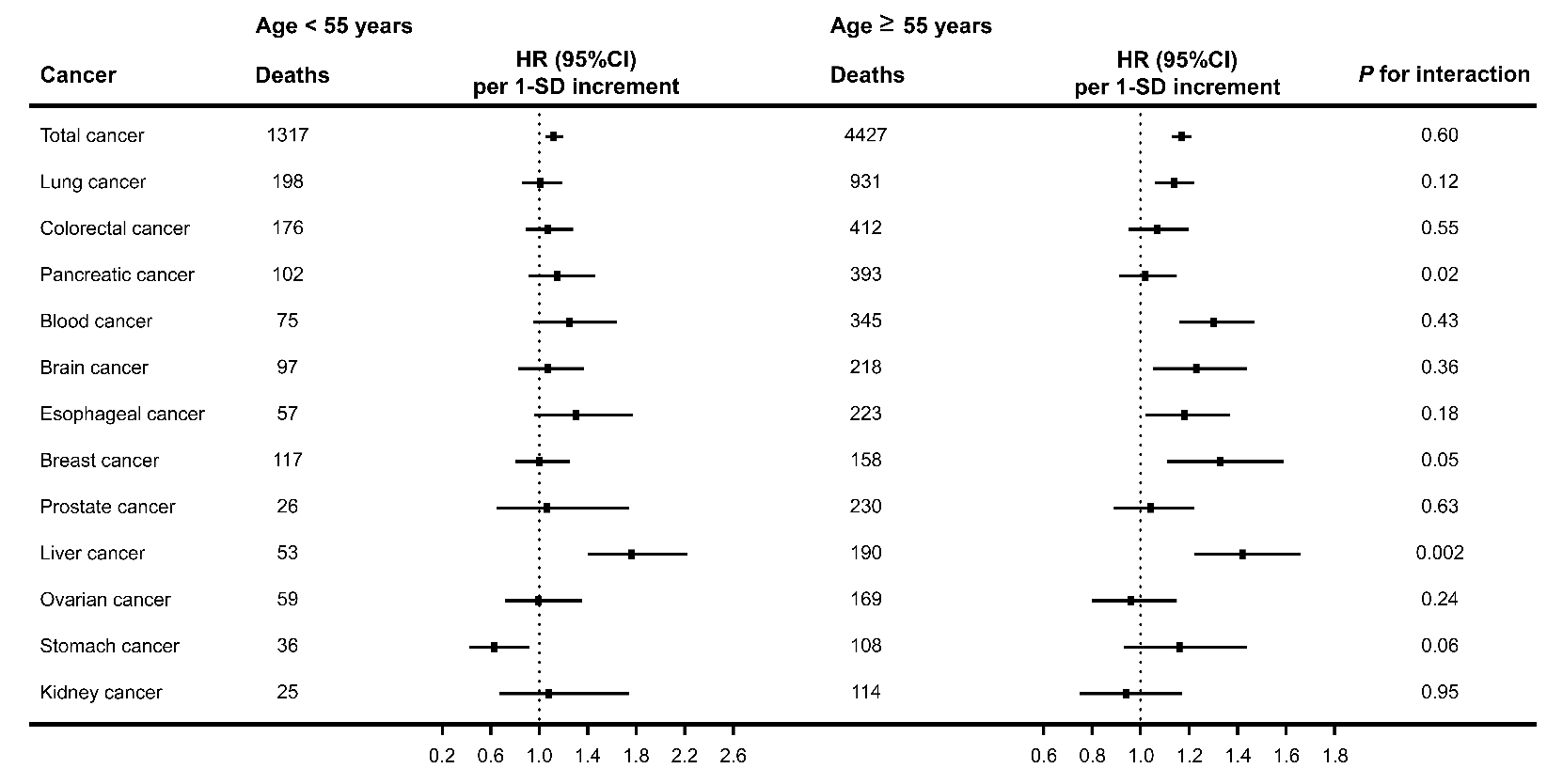


**Supplementary Figure 2.** Forest plots of stratified analyses of the associations between serum cystatin C concentrations and cancer-specific mortality according to age. HRs and 95% CIs for mortality were obtained in the fully-adjusted models. Round dots represent the HRs and whiskers represent the corresponding 95% CIs.

Analyses was adjusted for age at assessment (years), sex (female, male), ethnicity (white, not white) and fasting status (yes, no), Townsend deprivation index (continuous), college or university degree (yes, no), body mass index (kg/m^2^), smoking status (never, previous, current), pack years of smoking (continuous), alcohol consumption (never, special occasions only, 1-3 times per month, 1-2 times per week, 3-4 times per week, daily/almost daily), physical activity (MET-hours/week), family history of cancer (yes, no), prevalent hypertension (yes, no), prevalent diabetes (yes, no), prevalent cardiovascular diseases (yes, no), serum cholesterol (mmol/L), low-density lipoprotein (mmol/L), C-reactive protein concentrations (mg/L) and eGFR (ml/min/1.73m²), and in women menopausal status (yes, no) and ever use of hormone replacement therapy (yes, no). HR, hazard ratio; CI, confidence interval.


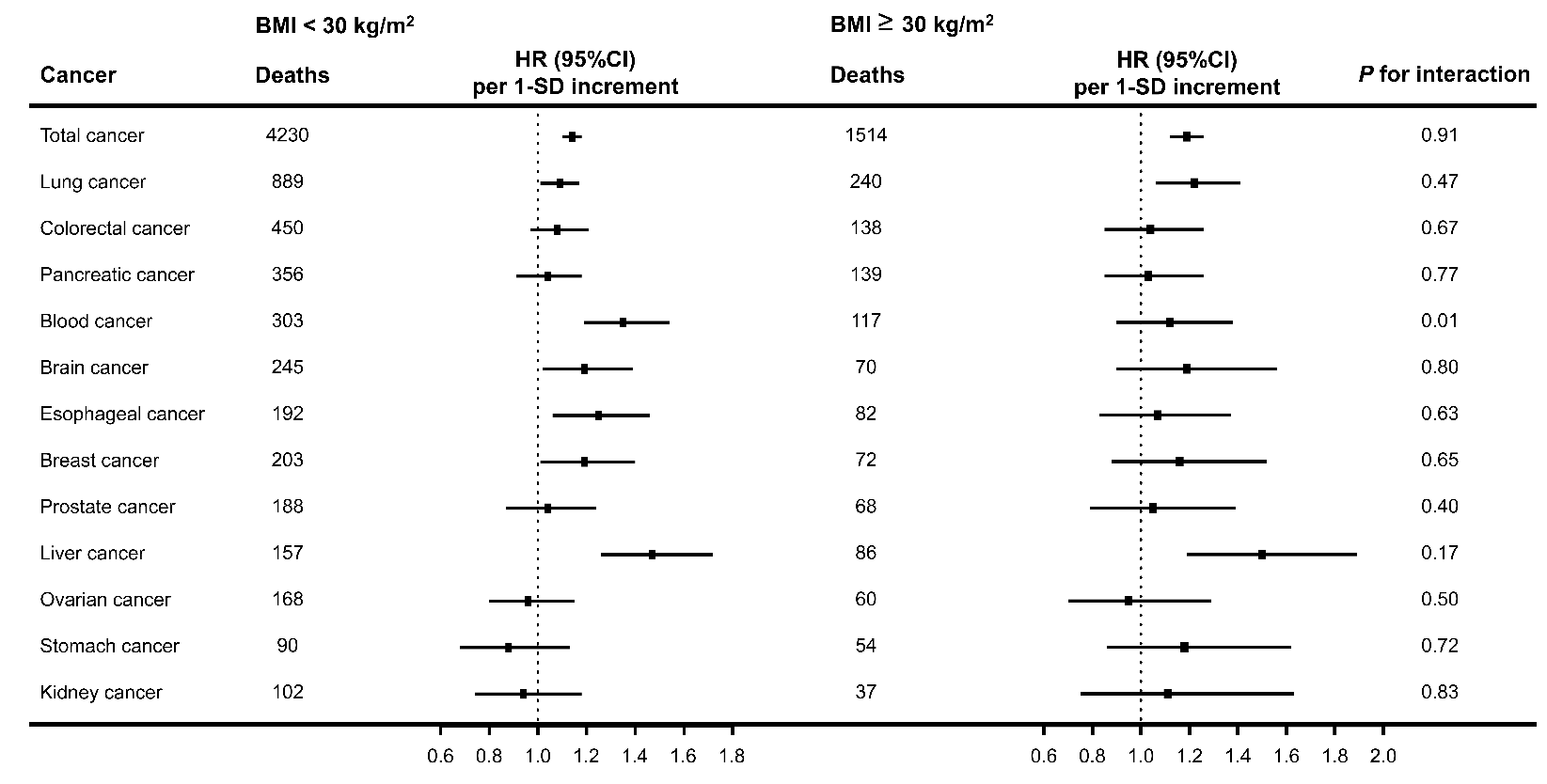


**Supplementary Figure 3.** Forest plots of stratified analyses of the associations between serum cystatin C concentrations and cancer-specific mortality according to sex. HRs and 95% CIs for mortality were obtained in the fully-adjusted models. Round dots represent the HRs and whiskers represent the corresponding 95% CIs.

Analyses was adjusted for age at assessment (years), sex (female, male), ethnicity (white, not white) and fasting status (yes, no), Townsend deprivation index (continuous), college or university degree (yes, no), body mass index (kg/m^2^), smoking status (never, previous, current), pack years of smoking (continuous), alcohol consumption (never, special occasions only, 1-3 times per month, 1-2 times per week, 3-4 times per week, daily/almost daily), physical activity (MET-hours/week), family history of cancer (yes, no), prevalent hypertension (yes, no), prevalent diabetes (yes, no), prevalent cardiovascular diseases (yes, no), serum cholesterol (mmol/L), low-density lipoprotein (mmol/L), C-reactive protein concentrations (mg/L) and eGFR (ml/min/1.73m²), and in women menopausal status (yes, no) and ever use of hormone replacement therapy (yes, no). HR, hazard ratio; CI, confidence interval.


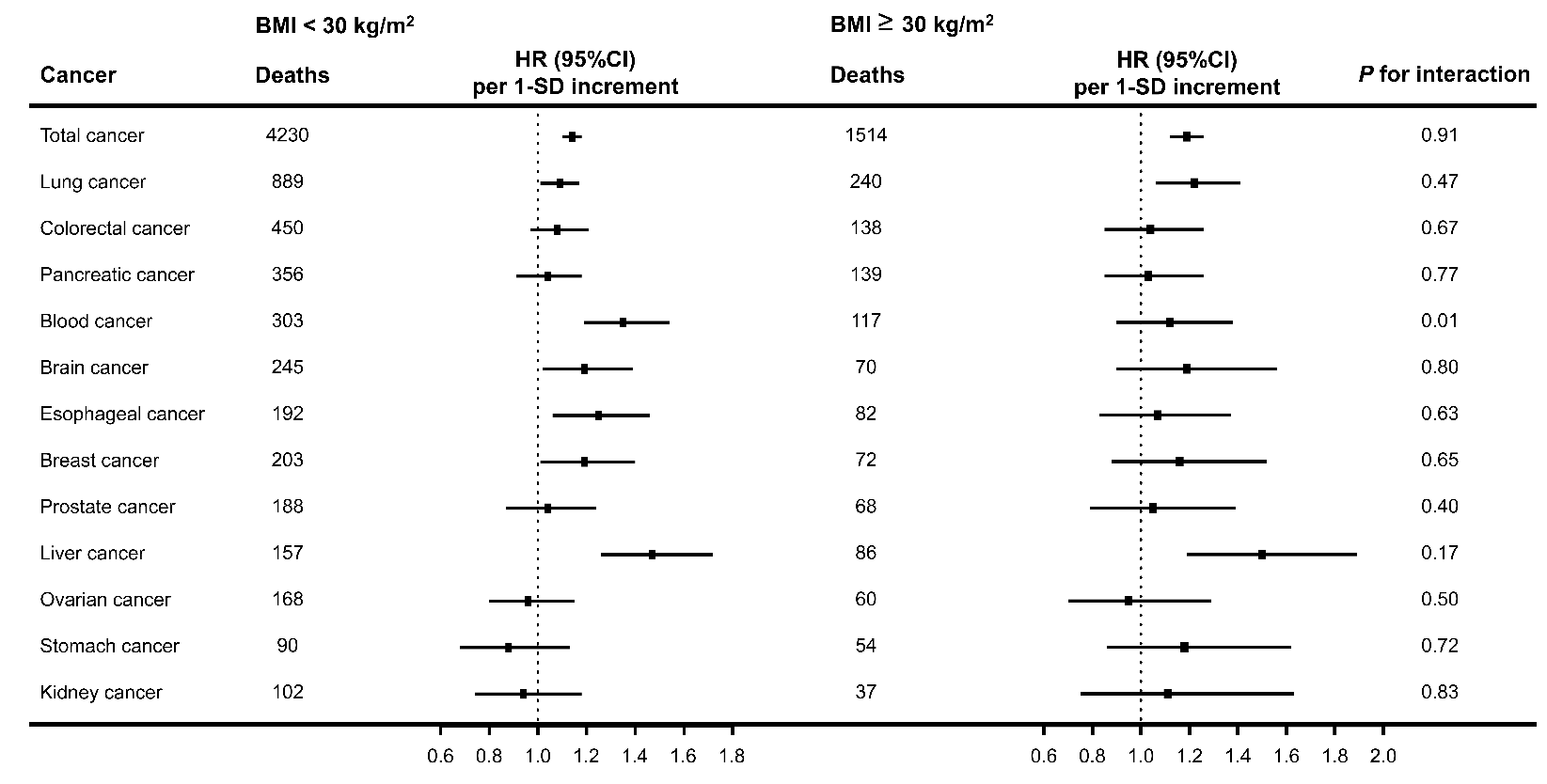


**Supplementary Figure 4.** Forest plots of stratified analyses of the associations between serum cystatin C concentrations and cancer-specific mortality according to BMI. HRs and 95% CIs for mortality were obtained in the fully-adjusted models. Round dots represent the HRs and whiskers represent the corresponding 95% CIs.

Analyses was adjusted for age at assessment (years), sex (female, male), ethnicity (white, not white) and fasting status (yes, no), Townsend deprivation index (continuous), college or university degree (yes, no), body mass index (kg/m^2^), smoking status (never, previous, current), pack years of smoking (continuous), alcohol consumption (never, special occasions only, 1-3 times per month, 1-2 times per week, 3-4 times per week, daily/almost daily), physical activity (MET-hours/week), family history of cancer (yes, no), prevalent hypertension (yes, no), prevalent diabetes (yes, no), prevalent cardiovascular diseases (yes, no), serum cholesterol (mmol/L), low-density lipoprotein (mmol/L), C-reactive protein concentrations (mg/L) and eGFR (ml/min/1.73m²), and in women menopausal status (yes, no) and ever use of hormone replacement therapy (yes, no). HR, hazard ratio; CI, confidence interval.


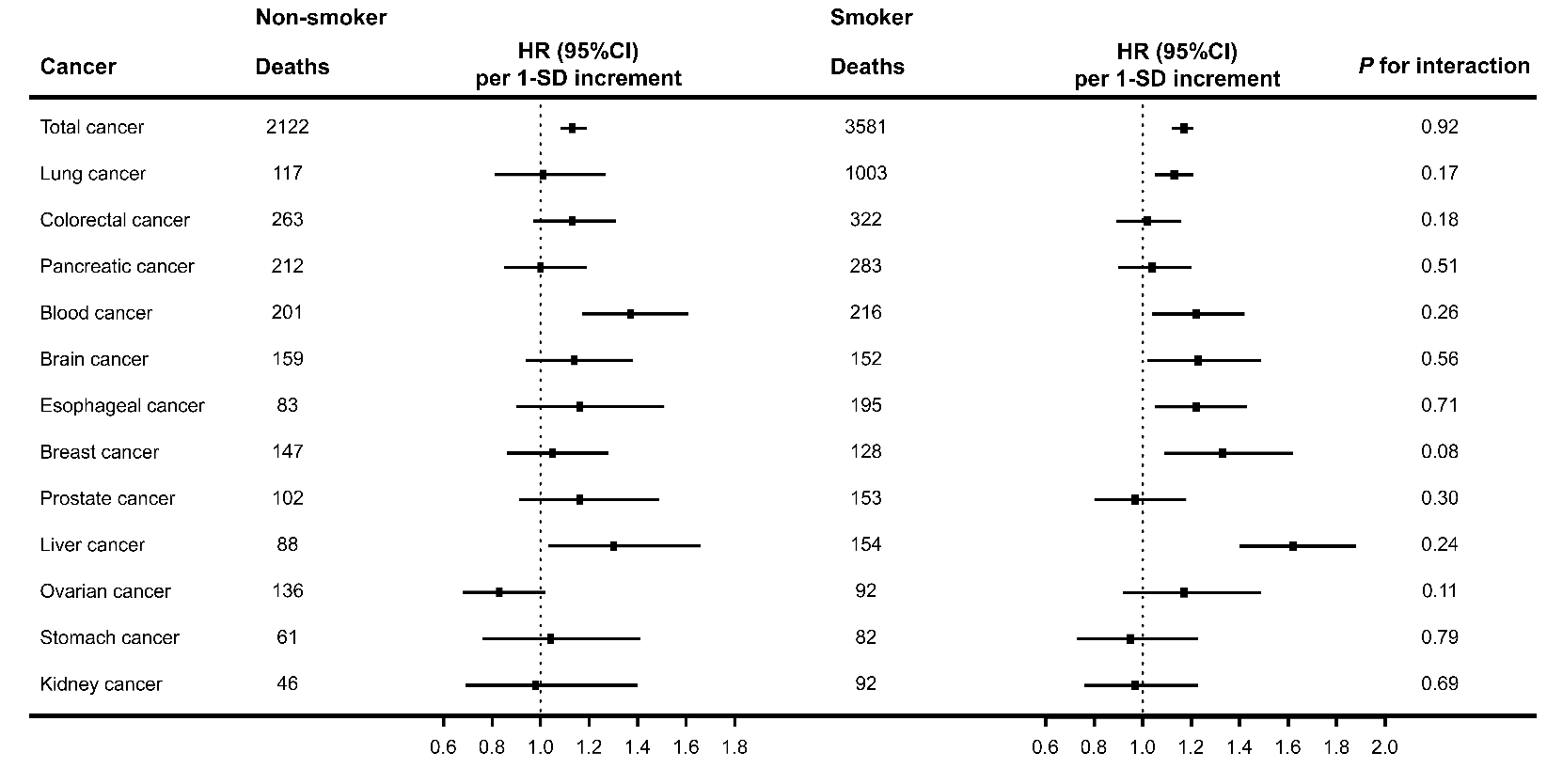


**Supplementary Figure 5.** Forest plots of stratified analyses of the associations between serum cystatin C concentrations and cancer-specific mortality according to smoking status. HRs and 95% CIs for mortality were obtained in the fully-adjusted models. Round dots represent the HRs and whiskers represent the corresponding 95% CIs.

Analyses was adjusted for age at assessment (years), sex (female, male), ethnicity (white, not white) and fasting status (yes, no), Townsend deprivation index (continuous), college or university degree (yes, no), body mass index (kg/m^2^), smoking status (never, previous, current), pack years of smoking (continuous), alcohol consumption (never, special occasions only, 1-3 times per month, 1-2 times per week, 3-4 times per week, daily/almost daily), physical activity (MET-hours/week), family history of cancer (yes, no), prevalent hypertension (yes, no), prevalent diabetes (yes, no), prevalent cardiovascular diseases (yes, no), serum cholesterol (mmol/L), low-density lipoprotein (mmol/L), C-reactive protein concentrations (mg/L) and eGFR (ml/min/1.73m²), and in women menopausal status (yes, no) and ever use of hormone replacement therapy (yes, no). HR, hazard ratio; CI, confidence interval.
